# Arthroscopic Fixation of Unstable Adolescent Knee Osteochondritis Dissecans With Structural Tibial Cancellous Bone Autograft

**DOI:** 10.1002/atn2.70261

**Published:** 2026-10-04

**Authors:** Yuta Tachibana, Tomoya Nishikawa, Tomoyuki Shimoji, Tomoki Ohori, Hiroyuki Tanaka, Seiji Okada, Kazutaka Kinugasa

**Affiliations:** ^1^ Department of Orthopaedic Sports Medicine Osaka Rosai Hospital Sakai‐City Osaka Japan; ^2^ Department of Orthopaedic Surgery The University of Osaka Graduate School of Medicine Suita‐City Osaka Japan

## Abstract

The treatment of knee osteochondritis dissecans in young patients remains challenging. Unstable or displaced lesions and those unresponsive to conservative management require surgical treatment. Because the articular surface morphology is complex and patient‐specific, preservation and fixation of the native osteochondritis dissecans fragment are ideal for maintaining anatomic congruity. Structural bone grafting is desirable in cases of unstable lesions with substantial subchondral bone loss because it provides effective defect filling, greater mechanical support for large defects, and resistance to postoperative lesion collapse. Arthroscopy offers several advantages over traditional open approaches through an arthrotomy, including reduced surgical invasiveness, improved visualization of intra‐articular pathology, and accelerated postoperative recovery. However, studies describing the optimal arthroscopic techniques are limited. Therefore, this article aims to describe a step‐by‐step arthroscopic technique of structural strip‐shaped tibial cancellous bone grafting for unstable adolescent knee osteochondritis dissecans lesions.

## Graphical abstract

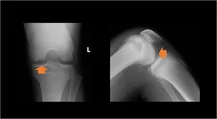

**VIDEO 1:** Technical note of arthroscopic fixation of unstable adolescent knee osteochondritis dissecans with structural tibial cancellous bone autograft. Careful probing is used to identify the unstable region of the lesion. In the classical osteochondritis dissecans, the lateral edge adjacent to the femoral attachment of the posterior cruciate ligament is frequently the most unstable. Conversely, the medial edge is stable and maintains continuity with the intact area of the medial femoral condyle. Accessory anterolateral working portals are created, and unstable soft tissue is thoroughly debrided using a mechanical shaver and curette. After debridement, the unstable edge of the lesion assumes a trapdoor. The anteromedial portal is extended to approximately 4 cm to allow smooth passage of the structural cancellous bone grafts without soft‐tissue entrapment. A cylindrical autologous bone graft with approximately 15 mm depth is harvested from the proximal medial tibia, and the cancellous portion of the graft is divided into strip‐shaped segments of approximately 3 mm thickness, carefully tailored to match the defect size in width and length. Under visualization through the anterolateral portal, the cancellous bone strips are introduced through the extended anteromedial incision and grasped with an arthroscopic forceps inserted through the far anterolateral portal. The bone grafts are then guided into the lesion bed using the arthroscopic forceps, whereas a tendon hook retractor inserted through the anteromedial incision gently elevates the trapdoor edge of the lesion and a standard arthroscopic hook introduced through the superior anterolateral portal assists with graft positioning. Each graft is advanced into the defect using a bone tamp. Internal fixation is then performed using multiple bioabsorbable pins. Under arthroscopic visualization through the anterolateral portal, the edge of the trapdoor lesion is closed using nylon sutures to prevent displacement of the implanted structural cancellous bone. Postoperative healing of the lesion is evaluated using computed tomography. Video content can be viewed at https://doi.org/10.1002/atn2.70261.

Knee osteochondritis dissecans (OCD) lesions are an uncommon cause of knee pain in adolescents, predominantly affecting active men aged 12 to 19 years, with an incidence of approximately 11.2 per 100,000 individuals.^1^ Approximately two‐thirds of the lesions were located on the medial femoral condyle.^2^ Surgical intervention is typically indicated for unstable or displaced lesions and those that do not respond to nonoperative treatment. Primary fixation of the native OCD fragment is the preferred option for restoring joint congruity because the articular surface morphology is highly patient‐specific. Traditionally, open reduction and fixation through an arthrotomy have been performed.^3^ Recently, arthroscopic approaches have gained popularity owing to their potential to minimize morbidity associated with open surgery, evaluate intra‐articular pathologies, reduce and fix the fragment more precisely, and accelerate postoperative recovery. Despite these advantages, arthroscopic bone grafting of the lesion bed is technically demanding. Structural bone grafting is desirable in cases of unstable lesions with substantial subchondral bone loss. However, studies describing the optimal arthroscopic techniques are limited. This article aims to describe the detailed technique of arthroscopic structural strip‐shaped tibial cancellous bone grafting for unstable adolescent knee OCD lesions.

## SURGICAL TECHNIQUE

### Preoperative Evaluation

A comprehensive medical history is obtained with particular attention to the patient's age and skeletal maturity. Preoperative imaging includes plain radiographs (Figure 1), computed tomography, and magnetic resonance imaging (Figures 2 and 3). These imaging modalities are used to assess the lesion size, location, and stability and physeal status.

**FIGURE 1:**
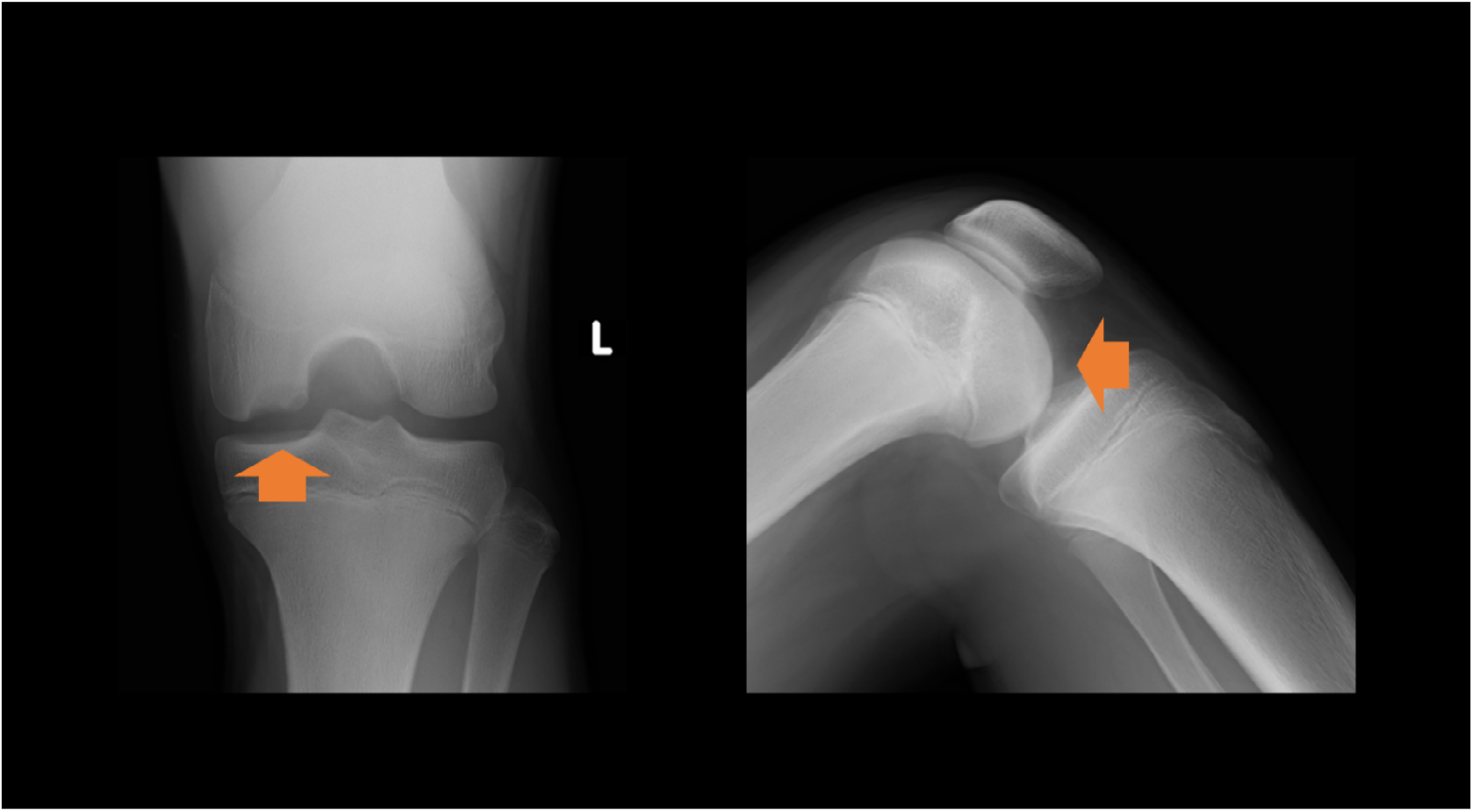
Preoperative plain radiographs of the left knee (left: Rosenberg view, right: lateral view). Orange arrows indicate a knee osteochondral dissecans of the MFC. (MFC, medial femoral condyle.)

**FIGURE 2:**
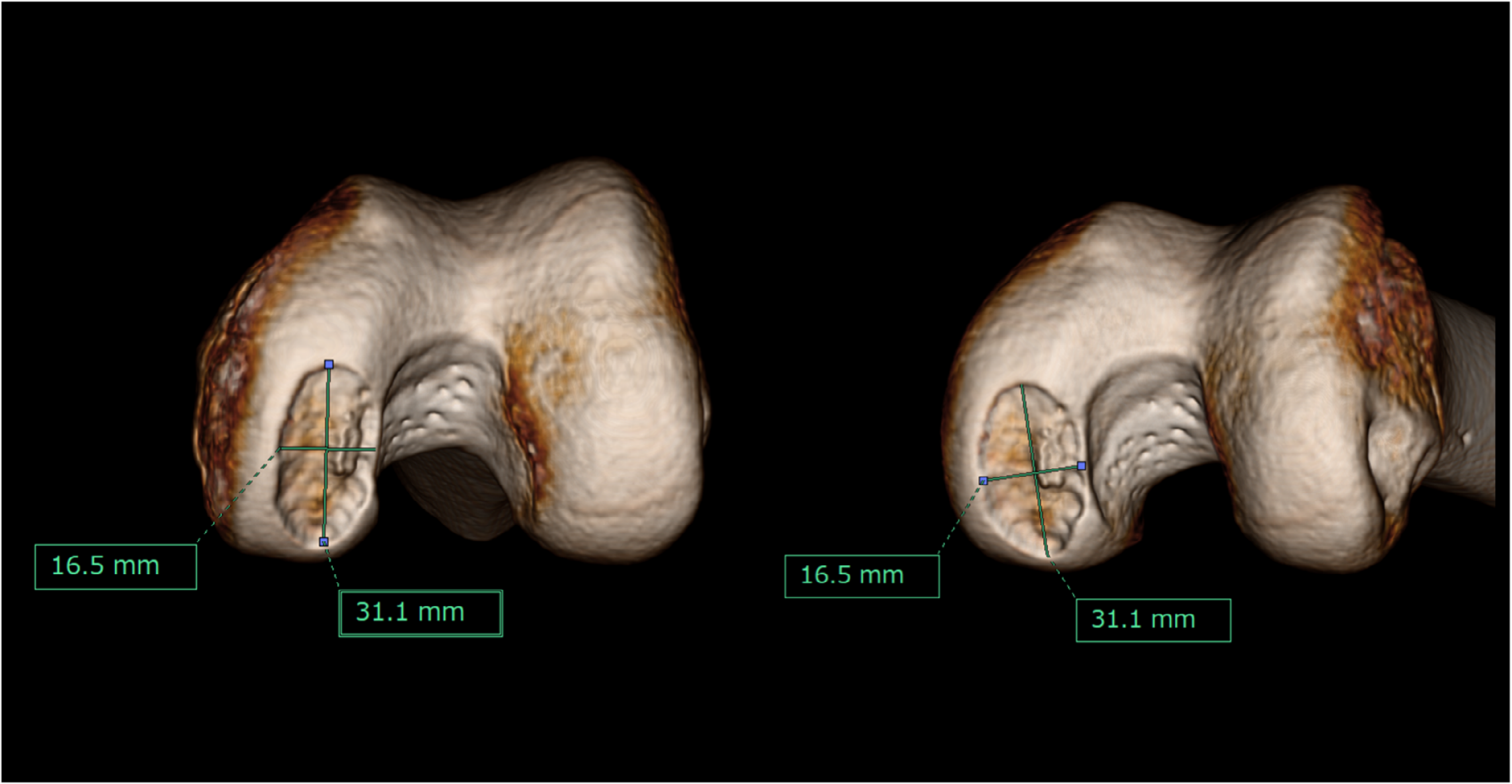
Preoperative CT images of the left knee. There is a substantial knee osteochondral dissecans of the MFC (>400 mm^2^). (CT, computed tomography; MFC, medial femoral condyle.)

**FIGURE 3:**
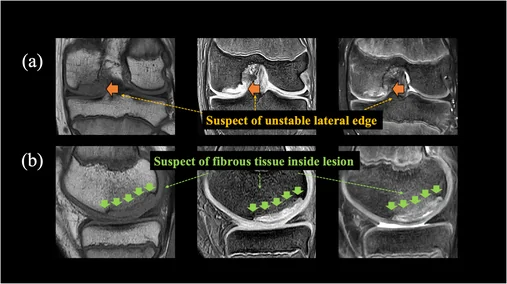
Preoperative MRI images of the left knee. (a) The lateral edge of the lesion adjacent to the femoral attachment of the PCL (orange arrows) is likely to be unstable in the coronal slices. (b) There is a suspected fibrous tissue inside the lesion (light green arrows) in the sagittal slices. (MRI, magnetic resonance imaging; PCL, posterior cruciate ligament.)

### Patient Positioning and Arthroscopic Assessment

Standard anterolateral and anteromedial portals are established for arthroscopic visualization, and careful probing is performed to identify the stable and unstable regions of the lesion. Classic medial femoral condyle OCD lesions are commonly located near the femoral attachment of the posterior cruciate ligament,^4^ and the lateral edge adjacent to that attachment is the most unstable, which is characterized by fluid infiltration or fibrous tissue continuity. Conversely, the medial edge is stable and maintains continuity with the intact area of the medial femoral condyle (Figure 4, Video 1). Accessory superior and far anterolateral portals are created as needed. Unstable fibrous tissue is debrided from those accessory anterolateral portals under arthroscopic visualization through the anterolateral portal (Figure 5a), and the bony bed is thoroughly refreshed to expose viable subchondral bone (Figure 5b). Particular care is taken to preserve the intact medial edge (hinge side) of the lesion and maintain continuity with the intact surrounding cartilage. The length, width, and depth of the osteochondral defect are arthroscopically measured after thorough refreshment of the bony bed.

**FIGURE 4:**
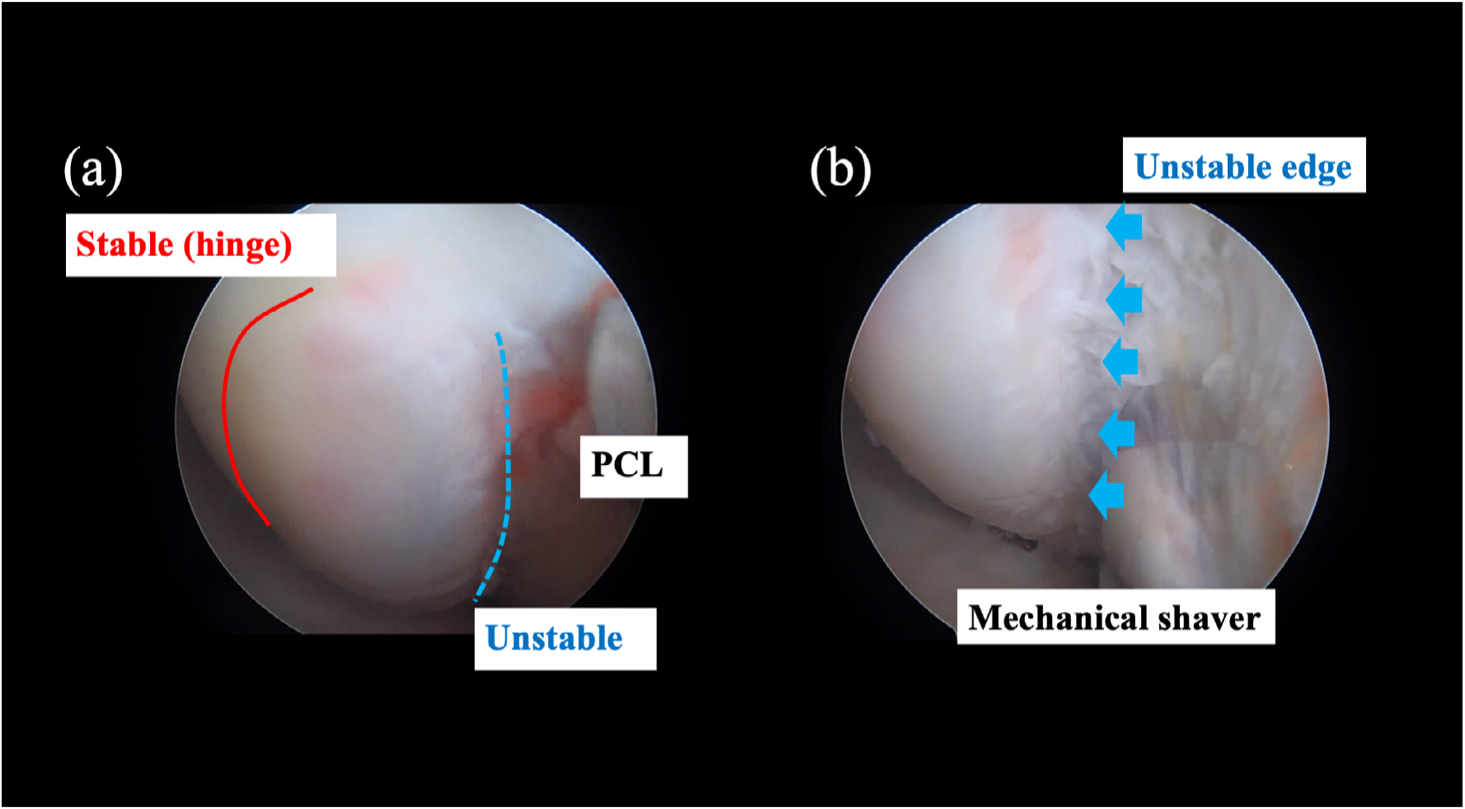
Arthroscopic view of the left knee under an anterolateral portal. The patient is set in a supine position with the knee flexed at 90°. (a) The lateral edge (light blue dashed line) adjacent to the femoral attachment of the PCL is the most unstable. Conversely, the medial edge (red solid line) is stable and maintains continuity with the intact area of the MFC. (b) Unstable fibrous tissues are carefully debrided from the unstable edge (light blue arrows) using accessory anterolateral portals. (PCL, posterior cruciate ligament; MFC, medial femoral condyle.)

**FIGURE 5:**
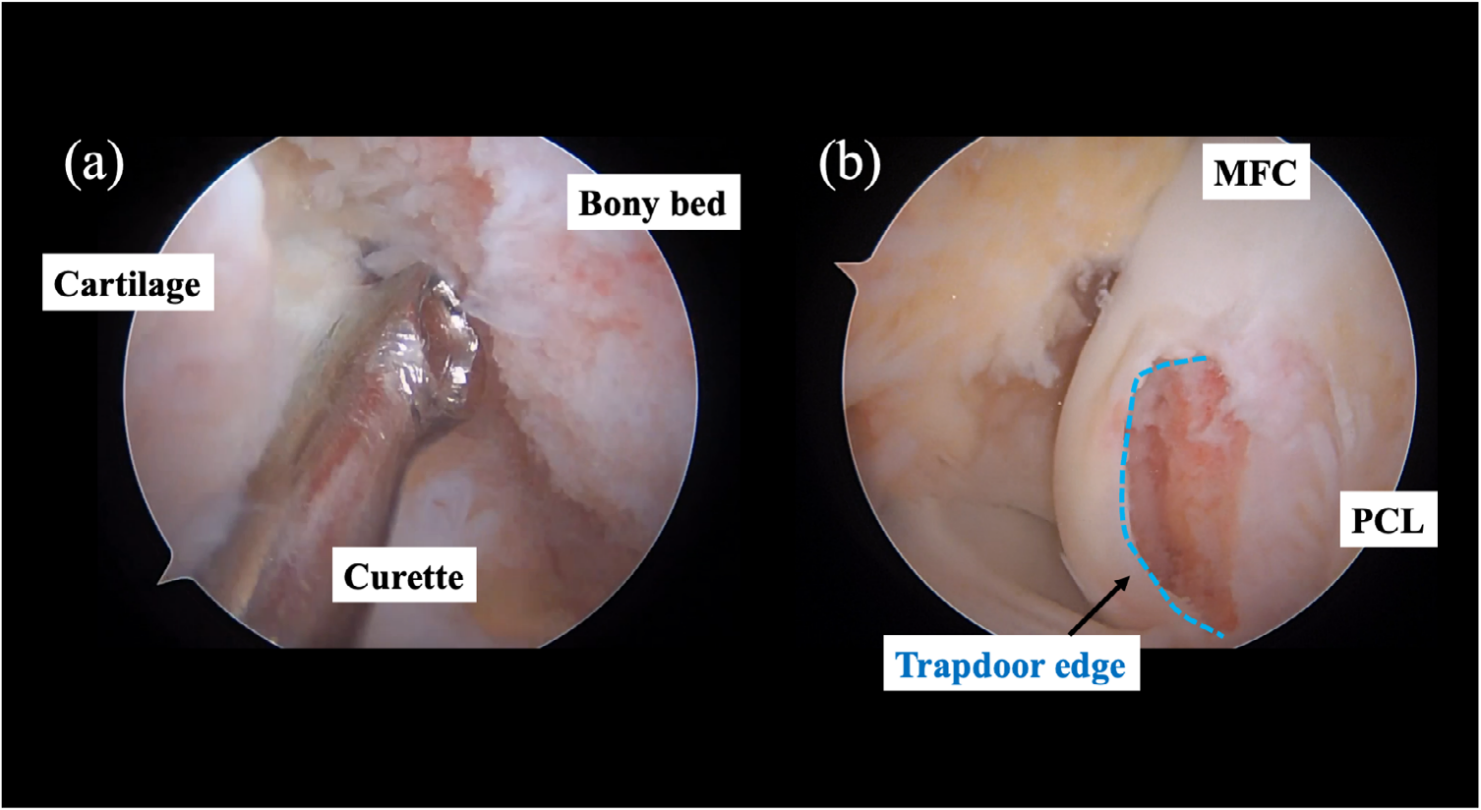
Arthroscopic view of the left knee under an anterolateral portal. (a) Unstable fibrous tissues are debrided from accessory anterolateral portals using a mechanical shaver and a curette. (b) After the bony bed is thoroughly refreshed to expose viable subchondral bone, the unstable edge of the lesion (light blue dashed line) resembles a trapdoor. (MFC, medial femoral condyle; PCL, posterior cruciate ligament.)

### Bone Graft Harvest and Preparation

A skin incision approximately 2 cm in length is made just proximal to the pes anserinus insertion. Blunt dissection of the subcutaneous tissue readily exposes the periosteal surface. In adolescent patients, the harvest site and trajectory are carefully planned based on preoperative computed tomography imaging to avoid physeal injury. Intraoperative fluoroscopic confirmation may be used to enhance the safety. A cylindrical autologous bone graft with approximately 15 mm depth is harvested from the proximal medial tibia using an osteochondral autograft harvesting system (SDS System, Zimmer Germany GmbH, Freiburg, Germany). The cancellous portion of the graft is divided into strip‐shaped segments of approximately 3 mm thickness, carefully tailored to match the defect size. The length and width of the strip‐shaped cancellous bones are also adjusted to match the osteochondral defect size. The donor site is filled with a cylindrical artificial bone substitute (OSferion 60; OSferionBiomaterials, Tokyo, Japan), sealed with a cortical portion of the graft, and covered with periosteum. The anteromedial portal is extended to approximately 4 cm to facilitate the smooth passage of the structural cancellous bone grafts without soft‐tissue entrapment (Figure 6).

**FIGURE 6:**
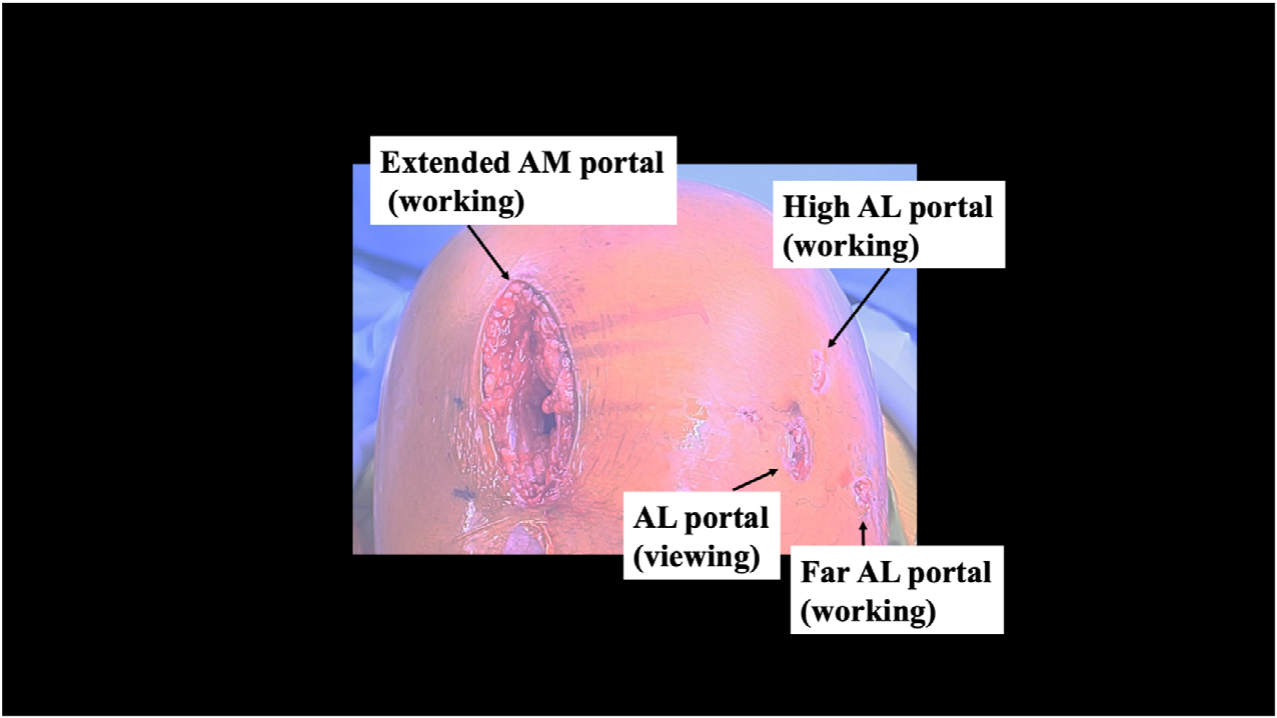
Skin incisions of the left knee. Extended AM portal, standard AL portal, and accessory far and high AL portals. (AL, anterolateral; AM, anteromedial.)

### Arthroscopic Structural Cancellous Bone Graft Insertion and Fixation

The cancellous bone strips are introduced through the extended anteromedial incision. Arthroscopic forceps are inserted through the far anterolateral portal to grasp and guide the bone grafts into the lesion bed (Figure 7). A tendon hook retractor is introduced through the anteromedial incision and gently elevates the trapdoor edge of the lesion, whereas a standard arthroscopic hook is inserted through the superior anterolateral portal to assist with graft positioning (Figure 8a). Each graft is advanced into the defect using a bone tamp (Figure 8b). This process is repeated until the subchondral defect is filled with structural cancellous bone.

**FIGURE 7:**
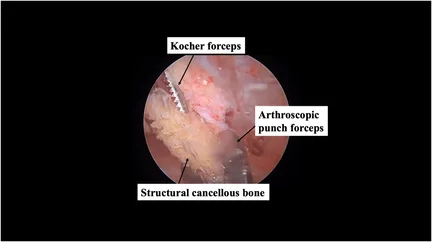
Arthroscopic view of the left knee under an AL portal. A structural tibial cancellous bone is passed through an extended AM portal and grasped by an arthroscopic forceps through a far AL portal. (AL, anterolateral; AM, anteromedial.)

**FIGURE 8:**
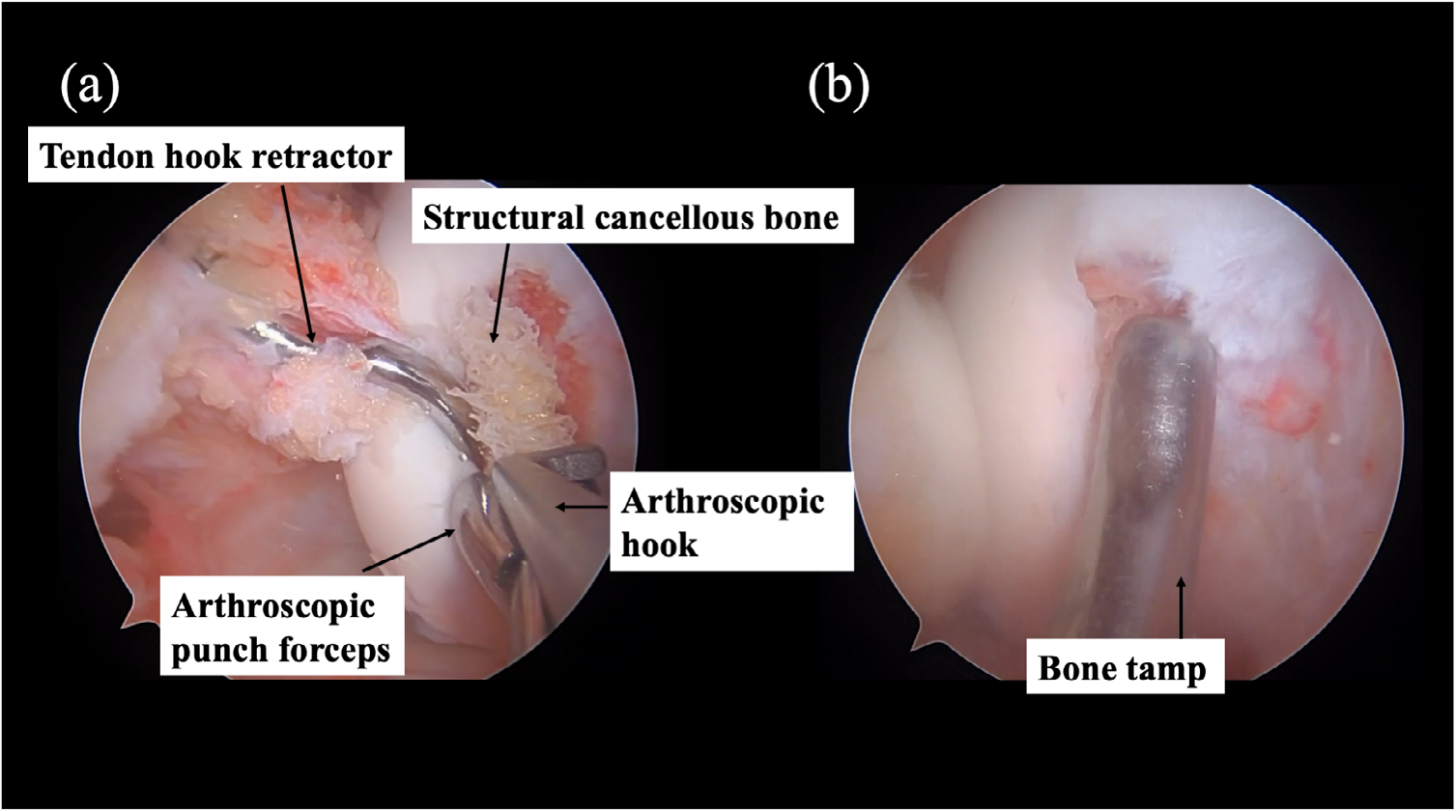
Arthroscopic view of the left knee under an AL portal. (a) A structural strip‐shaped tibial cancellous bone grasped by an arthroscopic forceps through a far AL portal is delivered into the bony bed. A tendon hook retractor is introduced through the AM incision and gently elevates the trapdoor edge of the lesion, whereas a standard arthroscopic hook is inserted through a high AL portal to assist with graft positioning. (b) A structural strip‐shaped tibial cancellous bone is gently placed by a bone tamp through a far AL portal. (AL, anterolateral; AM, anteromedial.)

Internal fixation is achieved using multiple bioabsorbable pins (GRAND FIX, Gunze Medical, Osaka, Japan) at approximately 5 mm intervals to ensure that they are evenly spaced. Under arthroscopic visualization through the anterolateral portal, the edge of the trapdoor lesion is closed using 5 to 0 nylon sutures (NESCOSUTURE, Alfresa Pharma, Osaka, Japan) to prevent displacement of the implanted structural cancellous bone. The use of a Hegar needle holder is helpful for facilitating suture passage by taking advantage of the needle curvature (Figure 9).

**FIGURE 9:**
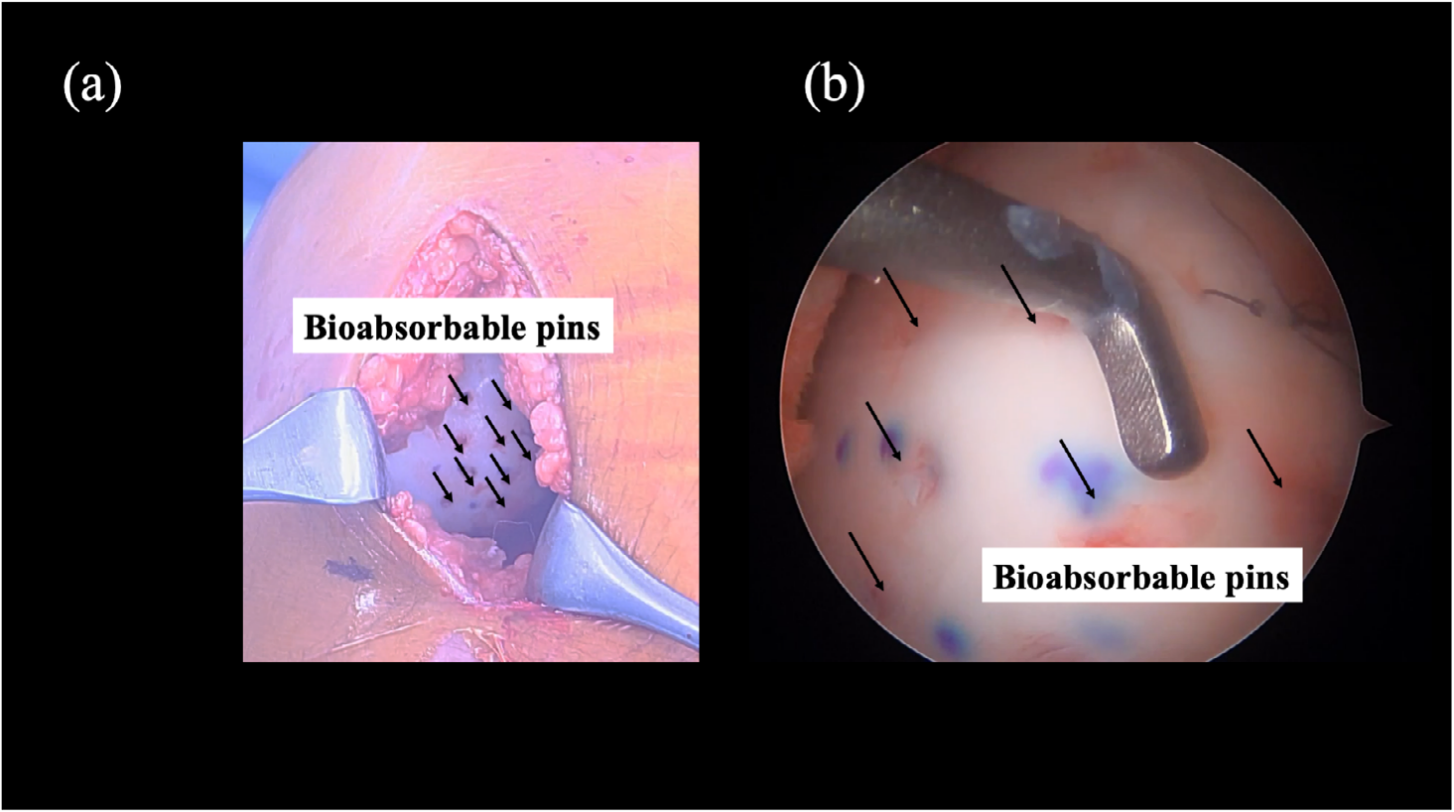
Findings of the lesion of the left knee under (a) macroscopic view through the AM incision and (b) arthroscopic view from an AL portal after fixation with multiple bioabsorbable pins (black arrows). (AL, anterolateral; AM, anteromedial.)

### Postoperative Protocol and Rehabilitation

The knee is immobilized in a brace for 2 weeks after surgery, after which range‐of‐motion exercises are initiated. Partial weight‐bearing is started at 4 weeks and full weight‐bearing at 6 weeks. Jogging is permitted approximately at 4 months, and jumping and sprinting are introduced at 6 months. Return to strenuous sports activity is allowed at 10 months. The postoperative healing status is evaluated using computed tomography (Figure 10).

**FIGURE 10:**
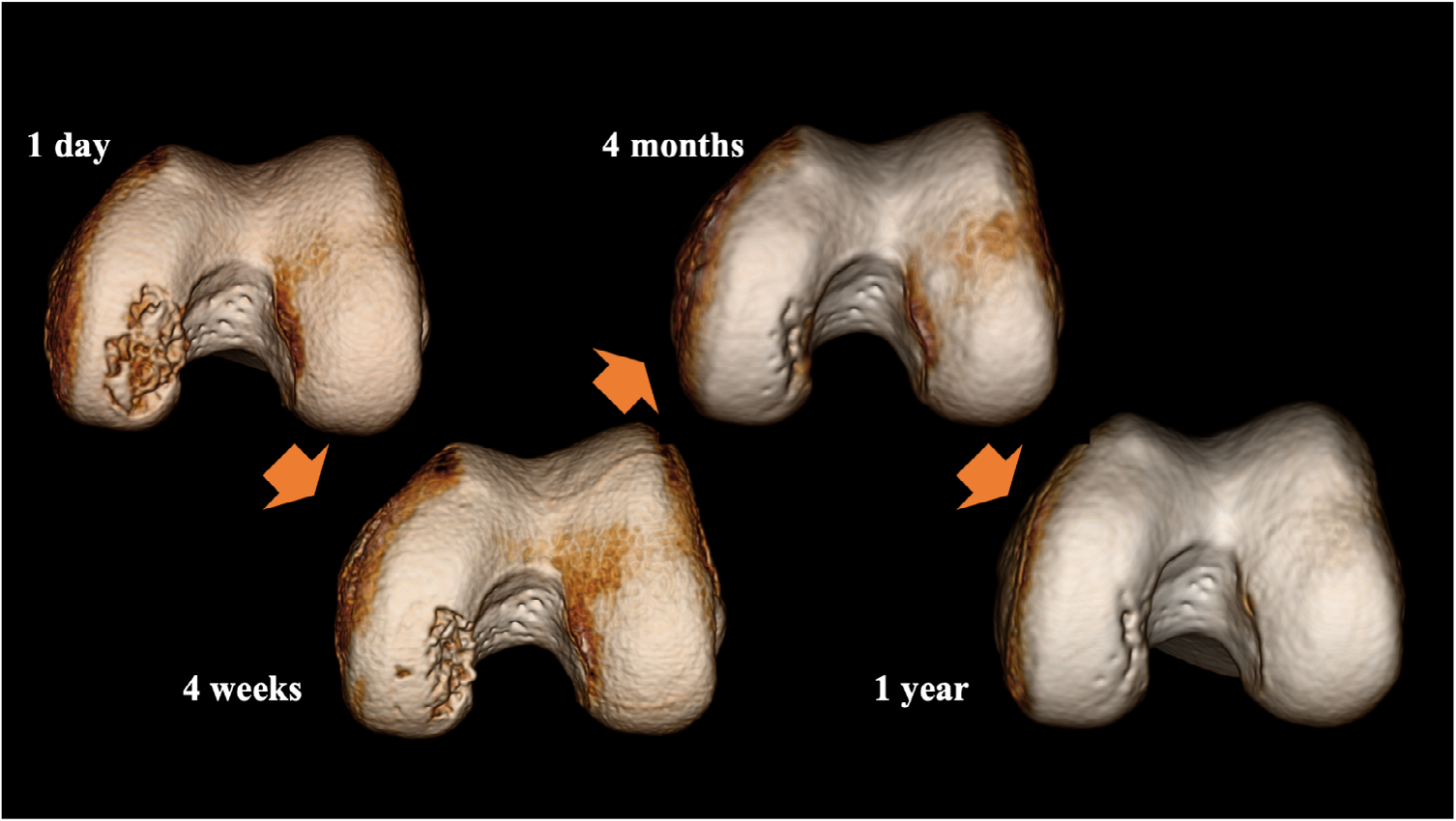
Postoperative CT images of the left knee. Bony integration is rapidly observed at 4 weeks and almost completed at 4 months. (CT, computed tomography.)

## DISCUSSION

This article describes an arthroscopic structural cancellous bone grafting technique combined with fragment fixation for unstable adolescent medial femoral condyle OCD lesions. The described technique is particularly suitable for unstable but nondisplaced lesions with substantial subchondral bone defects in which fragment preservation is feasible. Primary fixation of the OCD fragment is the preferred strategy because of the patient‐specific morphology of the articular surface and the relative rarity of severe cartilage degeneration in adolescents. A long‐term study has shown higher rates of osteoarthritis and knee arthroplasty after fragment excision than those after fragment preservation, highlighting the importance of restoring native osteochondral congruity.^5^ Surgical treatment aims to achieve lesion stability and promote healing, and autologous bone grafting is an ideal approach for the latter. Recently, several studies have reported arthroscopic bone grafting techniques using a transparent tuberculin syringe^6^ or suction tip^7^ after elevating the trapdoor edge and refreshing the bony bed.^8^ However, these methods may deliver only paste or piece cancellous bone grafting and carry the risk of graft leakage when the syringe or suction tip is used, which may lead to synovitis and loose body formation. Conversely, the use of structural cancellous bone grafts provides effective defect filling, greater mechanical support for large defects, and resistance to postoperative lesion collapse.

Several pearls should be considered when performing this technique (Table 1): (1) detecting the unstable side of the lesion and debriding the bony bed, (2) elevating the unstable side (trapdoor) of the lesion while maintaining the hinge side to avoid complete detachment of the fragment, (3) dividing cylindrical cancellous bone into structural strip‐shaped pieces matching the defect size, and (4) extending the anteromedial portal to smoothly pass structural cancellous bones.

**TABLE 1:** Pearls and Pitfalls

Pearls
Preserve the hinge side of the lesion and maintain cartilage congruity
Create accessory superior and far anterolateral working portals for bony bed debridement and bone graft placement
Extend the anteromedial portal to facilitate the delivery of structural cancellous bone

Pitfalls
Convert to an open procedure if arthroscopic skills are insufficient or the hinge is compromised
Potential inflammatory reaction or hardware backout with bioabsorbable implants

Despite its advantages, the described technique has some limitations (Table 2). Arthroscopic bone grafting is technically demanding^6^, ^7^ and may require a longer operative time. Surgeons should consider converting to an open approach if arthroscopic skills are insufficient or the hinge is compromised. Paste or piece cancellous bone grafting techniques may be more appropriate for smaller lesions (<15 mm).^6^, ^7^

**TABLE 2:** Advantages and Disadvantages

Advantages
Minimally invasive approach
Precise intra‐articular assessment and treatment under arthroscopy
Structural cancellous bone graft resistance to postoperative lesion collapse
Preservation of native cartilage continuity

Disadvantages
Technical complexity
Potentially longer operative time

## DISCLOSURES

The authors (Y.T., T.N., T.S., T.O., H.T., S.O., K.K.) declare that they have no known competing financial interests or personal relationships that could have appeared to influence the work reported in this article.
